# Systemic inflammation response index is a useful indicator in distinguishing MOGAD from AQP4-IgG-positive NMOSD

**DOI:** 10.3389/fimmu.2023.1293100

**Published:** 2024-01-08

**Authors:** Lei Wang, Ruihong Xia, Xiangliang Li, Jingli Shan, Shengjun Wang

**Affiliations:** Department of Neurology, Qilu Hospital, Cheeloo College of Medicine, Shandong University, Jinan, Shandong, China

**Keywords:** myelin oligodendrocyte glycoprotein antibody-associated disease (MOGAD), neuromyelitis optica spectrum disorders (NMOSD), systemic inflammation response index (SIRI), monocyte-to-lymphocyte ratio (MLR), cerebrospinal fluid (CSF)

## Abstract

**Objective:**

To identify reliable immune-inflammation indicators for distinguishing myelin oligodendrocyte glycoprotein antibody-associated disease (MOGAD) from anti–aquaporin-4 immunoglobulin G (AQP4-IgG)-positive neuromyelitis optica spectrum disorders (NMOSD). To assess these indicators’ predictive significance in MOGAD recurrence.

**Methods:**

This study included 25 MOGAD patients, 60 AQP4-IgG-positive NMOSD patients, and 60 healthy controls (HCs). Age and gender were matched among these three groups. Participant clinical and imaging findings, expanded disability status scale (EDSS) scores, cerebrospinal fluid (CSF) information, and blood cell counts were documented. Subsequently, immune-inflammation indicators were calculated and compared among the MOGAD, AQP4-IgG-positive NMOSD, and HC groups. Furthermore, we employed ROC curve analysis to assess the predictive performance of each indicator and binary logistic regression analysis to assess potential risk factors.

**Results:**

In MOGAD patients, systemic inflammation response index (SIRI), CSF white cell count (WCC), and CSF immunoglobulin A (IgA) levels were significantly higher than in AQP4-IgG-positive NMOSD patients (p = 0.038, p = 0.039, p = 0.021, respectively). The ROC curves showed that SIRI had a sensitivity of 0.68 and a specificity of 0.7 for distinguishing MOGAD from AQP4-IgG-positive NMOSD, with an AUC of 0.692 (95% CI: 0.567-0.818, p = 0.0054). Additionally, compared to HCs, both MOGAD and AQP4-IgG-positive NMOSD patients had higher neutrophils, neutrophil-to-lymphocyte ratio (NLR), SIRI, and systemic immune-inflammation index (SII). Eight (32%) of the 25 MOGAD patients had recurrence within 12 months. We found that the monocyte-to-lymphocyte ratio (MLR, AUC = 0.805, 95% CI = 0.616–0.994, cut-off value = 0.200, sensitivity = 0.750, specificity = 0.882) was an effective predictor of MOGAD recurrence. Binary logistic regression analysis showed that MLR below 0.200 at first admission was the only risk factor for recurrence (p = 0.005, odds ratio =22.5, 95% CI: 2.552–198.376).

**Conclusion:**

Elevated SIRI aids in distinguishing MOGAD from AQP4-IgG-positive NMOSD; lower MLR levels may be linked to the risk of MOGAD recurrence.

## 1 Introduction

Myelin oligodendrocyte glycoprotein antibody-associated disease (MOGAD) can occur in individuals across all age groups, with an incidence rate of 1.6-3.4 per million and a prevalence of 20 per million (1, 2). The application of cell-based assays (CBA) has clarified MOGAD diagnoses for patients who were previously categorized as anti–aquaporin-4 immunoglobulin G (AQP4-IgG)-negative neuromyelitis optica spectrum disorders (NMOSD) (3). These patients often present with optic neuritis, longitudinally extensive transverse myelitis, or related conditions. Despite its rarity, research on MOGAD remains in its nascent stages. The clinical and radiological features of both MOGAD and AQP4-IgG-positive NMOSD exhibit some overlap, creating challenges for early diagnosis. A UK study with a median follow-up of 15.5 months found that 27% of MOGAD patients experienced recurrence, and 47% suffered lasting neurological deficits (4). Thus, it is crucial to identify indicators that can early distinguish MOGAD from AQP4-IgG-positive NMOSD and predict recurrence in MOGAD.

Systemic inflammation response index (SIRI), a marker of systemic inflammation calculated as the product of monocyte and neutrophil-to-lymphocyte ratio (NLR), has demonstrated its predictive power in various conditions like ischemic stroke, aneurysmal subarachnoid hemorrhage, glioma, and rheumatoid arthritis (5–8). However, its role in predicting MOGAD has not been explored.

Previous investigations have emphasized monocytes as significant contributors in demyelination (9–11). In AQP4-IgG-positive NMOSD mice, promoting neutrophil apoptosis showed potential for reducing brain damage (12). Early-stage inhibition of peripheral blood platelet elevation was linked to halting disease progression (13). Furthermore, AQP4-IgG-positive NMOSD patients exhibited significantly elevated neutrophils, NLR, platelet-to-lymphocyte ratio (PLR), and platelet × NLR (systemic immune-inflammation index, SII) compared to both multiple sclerosis (MS) patients and healthy individuals (14). Additionally, the monocyte-to-lymphocyte ratio (MLR) was effective in predicting NMOSD recurrence (14). Moreover, the expression of MOG protein is generally limited to the nervous system (15), unlike AQP4, which is widely distributed throughout the human body (16). This implies differences in CSF white cell count (WCC) and immunoglobulin between MOGAD and AQP4-IgG-positive NMOSD. Building upon these findings, we propose that these immune-inflammatory indicators could potentially assist in the early differentiation of MOGAD from AQP4-IgG-positive NMOSD and recurrence prediction of MOGAD.

## 2 Method

### 2.1 Study population

This study included 25 patients diagnosed with MOGAD and 60 patients with age- and sex-matched AQP4-IgG-positive NMOSD at Qilu Hospital, Cheeloo College of Medicine, Shandong University, from January 2018 to August 2022. Inclusion criteria were as follows: (1) Serum MOG antibody positivity detected through CBA and meeting the 2023 International MOGAD Panel proposed criteria (17); (2) AQP4-IgG-positive NMOSD diagnosis according to the 2015 International Panel for NMO Diagnosis (IPND) criteria (18); (3) All MOGAD and AQP4-IgG-positive NMOSD patients admitted to our hospital within 2 weeks of symptom onset; (4) No symptoms of systemic infection (like respiratory, urinary, skin, or soft tissue infections) or recent corticosteroid/immunosuppressive therapy within 14 days before blood collection; (5) Comprehensive clinical evaluations and laboratory assessments (including anti-double-stranded DNA antibodies, anti-SS-A and anti-SS-B antibodies, etc.) revealed no coexistence with hematological disorders or other autoimmune diseases; (6) Ages 14 and older. Exclusion criteria included: (1) Loss of follow-up, which referred to patients no longer returning for outpatient visits and couldn’t be reached by phone; (2) Inability to comply with neurologist-prescribed ongoing treatment. We also included 60 healthy individuals matched for gender and age with MOGAD patients as a control group; all were recruited from the Health Examination Center of Qilu Hospital, Shandong University.

### 2.2 Data collection

Upon admission, fasting blood samples were collected to evaluate neutrophils, monocytes, lymphocytes, and platelets. And then, calculations were calculated for NLR, MLR, PLR, SIRI, and SII. In addition to this, we gathered data on CSF, including WCC, proteins, and the levels of immunoglobulins A (IgA), G (IgG), and M (IgM). Neurologists determined Extended Disability Status Scale (EDSS) scores in MOGAD and AQP4-IgG-positive NMOSD patients to assess disability. For each MOGAD patient, we conducted follow-ups at three and twelve months after their discharge to assess recurrence. Clinical recurrence refers to the emergence of new neurological deficits more than one month after the initial attack (17).

### 2.3 MOG-IgG and AQP4-IgG assay

MOG-IgG and AQP4-IgG were detected by means of commercial CBA (Euroimmun, Lübeck, Germany) according to the manufacturer’s recommendations. CBA using HEK-293 cells stably transfected with human full-length MOG and human AQP4, and then MOG-IgG and AQP4-IgG were detected using anti-human IgG (Fc) secondary antibodies and subsequently visualized by microscopy.

### 2.4 Data analysis

Statistical analysis utilized SPSS 26.0 and GraphPad Prism 8.0. The normality of continuous variables was determined using the Shapiro-Wilk test. For non-normally distributed continuous data, we presented the results as the median with the interquartile range (IQR). Comparisons between two-group variables were conducted by the Mann–Whitney U test, and the Kruskal-Wallis H test was used in three-group comparisons, with *post hoc* tests conducted using the Bonferroni method. We used the Chi-square test for categorical variables and conducted *post hoc* Chi-square tests for multiple comparisons. Receiver operating characteristic (ROC) curves evaluated the predictive ability of each index. The optimal cut-off value was calculated using the Youden test. Binary logistic regression analysis was used to determine risk factors.

## 3 Results

### 3.1 Baseline characteristics

The analysis included 25 MOGAD patients with a median age of 27 (15, 35), of whom 40% were female. The median EDSS score before treatment was 3.0 (2.0, 4.5) (**Table 1**). The major symptoms were cerebral monofocal or polyfocal deficits, accounting for 44% (n = 11). In addition, cerebral cortical encephalitis, often with seizures, was detected in 28% of cases (n = 7). Brainstem deficits were found in 6 cases (24%). Optic neuritis (ON) occurred in 4 cases, making up 16% of all cases. Acute disseminated encephalomyelitis was observed in 3 cases (12%), and cerebellar deficits were presented in 2 cases (8%). Among 60 AQP4-IgG-positive NMOSD patients, the most common was acute transverse myelitis, found in 35 cases (58.3%), followed by cerebral syndrome in 15 cases (25.0%). Additionally, area postrema syndrome occurred in 18 cases (30%), while ON was present in 13 cases (21.7%). Diencephalic syndrome was observed in 4 cases (6.7%). Within 12 months after discharge, 32% (n = 8) of MOGAD patients experienced recurrence, with a median time to recurrence of 3 (2, 9.75) months and a median EDSS score of 3.5 (2.0, 4.5) at recurrence. The most common recurrent symptoms were cerebral monofocal or polyfocal deficits, seen in 5 cases (62.5%). ON and brainstem deficits each occurred in 2 cases (25%), while myelitis was observed in 1 case (12.5%).

**Table 1:** Comparison of immune-inflammation indicators among the three groups.

Variables	MOGAD (n=25)	NMOSD (n=60)	HC (n=60)	P1	P2	P3
Age, median, (IQR)	27 (15,35)	25 (19,35)	28 (17,35)	>0.999	>0.999	>0.999
Female (n%)	10 (40)	30 (50)	25 (42)	0.400	0.887	0.360
Relapse (n%)	8 (32)	15 (25)	–	0.508	–	–
EDSS, median, (IQR)	3.0 (2.0,4.5)	3.5 (3.0,6.0)	–	0.393	–	–
NEU, median, (IQR), 10^9^/L	6.53 (5.19,11.65)	5.47 (4.02,7.63)	3.12 (2.57,4.04)	0.155	**<0.001**	**<0.001**
LYM, median, (IQR), 10^9^/L	1.85 (1.00,2.50)	1.87 (1.27,2.38)	1.98 (1.49,2.47)	>0.999	>0.999	0.826
MON, median, (IQR), 10^9^/L	0.41 (0.35,0.63)	0.44 (0.28,0.62)	0.40 (0.31,0.49)	>0.999	0.356	0.772
PLT, median, (IQR), 10^9^/L	283 (240,304)	258 (223,302)	250 (202,293)	0.500	0.067	0.718
NLR, median, (IQR)	3.66 (2.40,8.58)	2.93 (1.72,5.50)	1.66 (1.14,2.48)	0.454	**<0.001**	**<0.001**
MLR, median, (IQR)	0.27 (0.18,0.39)	0.20 (0.16,0.30)	0.18 (0.14,0.30)	0.259	0.057	>0.999
SIRI, median, (IQR), 10^9^/L	1.74 (1.04,4.40)	1.07 (0.69,1.89)	0.61 (0.39,1.04)	**0.038**	**<0.001**	**<0.001**
PLR, median, (IQR)	138.29 (109.48,273.79)	138.59 (92.56,215.87)	115.32 (103.92,143.73)	>0.999	0.051	0.172
SII, median, (IQR), 10^9^/L	1054.62 (601.58,2493.39)	702.72 (449.21,1512.26)	381.74 (272.88,577.00)	0.225	**<0.001**	**<0.001**
Imaging profile						
Cerebrum (without cortex) (n%)	13 (52)	16 (27)	–	**0.025**	–	–
Cerebral cortex (n%)	8 (32)	0	–	**<0.001**	–	–
Optic nerve (n%)	4 (16)	13 (22)	–	0.552	–	–
Diencephalon (n%)	2 (8)	4 (7)	–	>0.999	–	–
Pons (n%)	6 (24)	6 (10)	–	0.168	–	–
Medulla (n%)	1 (4)	19 (32)	–	**0.006**	–	–
Cerebellum (n%)	3 (12)	1 (2)	–	0.074	–	–
Cervical spinal cord (n%)	3 (12)	30 (50)	–	**0.001**	–	–
Thoracic spinal cord (n%)	1 (4)	21 (35)	–	**0.003**	–	–
Lumbar spinal cord (n%)	0	1	–	>0.999	–	–
CSF profile						
Proteins, median, (IQR), g/L	0.40 (0.28,0.52)	0.30 (0.23,0.46)	–	0.056	–	–
WCC, median, (IQR), 10^6^/L	20.5 (1.5,72.8)	4.0 (0.2,11.0)	–	**0.039**	–	–
IgG, median, (IQR), mg/L	35.50 (28.40,47.20)	31.85 (20.50,46.65)	–	0.156	–	–
IgA, median, (IQR), mg/L	5.06 (3.73,6.41)	2.58 (1.79,6.16)	–	**0.021**	–	–
IgM, median, (IQR), mg/L	0.62 (0.37,1.56)	0.45 (0.20,0.85)	–	0.070	–	–
Treatment						
Steroid hormone (n%)	17 (68)	44 (73)	–	0.619	–	–
Steroid hormone + IVIG (n%)	6 (24)	9 (15)	–	0.321	–	–
Steroid hormone + MMF (n%)	0	3 (5)	–	0.552	–	–
Steroid hormone + IVIG + MMF (n%)	2 (8)	1 (2)	–	0.206	–	–
Steroid hormone + IVIG + CYP (n%)	0	2 (3)	–	>0.999	–	–
Inebilizumab (n%)	0	1 (2)	–	>0.999	–	–

NEU, neutrophil; LYM, lymphocyte; MON, monocyte; PLT, platelet; NLR, neutrophil to lymphocyte ratio; MLR, monocyte to lymphocyte ratio; PLR, platelet to lymphocyte ratio; SIRI, systemic inflammation response index, monocyte × NLR; SII, systemic immune inflammation index, platelet × NLR; EDSS, Expanded Disability Status Scale; CSF, cerebrospinal fluid; WCC, white cell counts; IgG, immunoglobulin G; IgA, immunoglobulin A; IgM, immunoglobulin M; IVIG, intravenous immunoglobulin; MMF, mycophenolate mofetil; CYP, cyclophosphamide;

P1, Comparative findings between MOGAD and AQP4-IgG-positive NMOSD. P2, Comparative outcomes between MOGAD and HC. P3, Comparative analysis between AQP4-IgG-positive NMOSD and HC; bold, p<0.05.

### 3.2 Differences in immune-inflammation indicators among three groups

**Figure 1:**
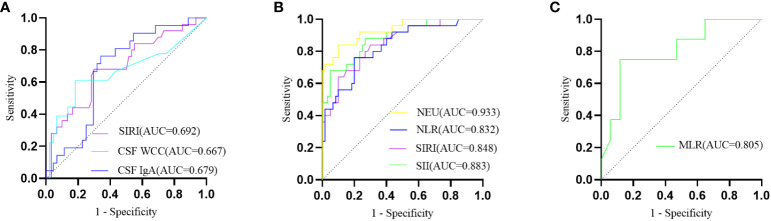
**(A)** The ROC curve depicting the discriminative prowess of SIRI, CSF WCC, and CSF IgA in differentially diagnosing between MOGAD and AQP4-IgG-positive NMOSD; **(B)** The ROC curve analysis delineating MOGAD from HC; **(C)** The ROC curve illustrating the predictive potential of MLR for MOGAD recurrence.

**Figure 2:**
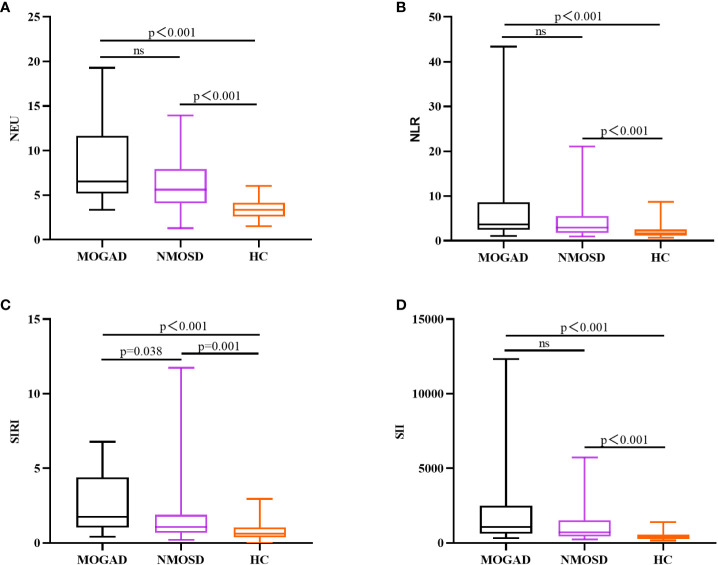
Box plots (with a median line at the center, a box representing the interquartile range, and whiskers extending from maximum to minimum) are used to elucidate the distributions of NEU **(A)**, NLR **(B)**, SIRI **(C)**, and SII **(D)** among MOGAD, AQP4-IgG-positive NMOSD, and HC.

In comparison to AQP4-IgG-positive NMOSD, MOGAD exhibited greater levels of SIRI, CSF WCC, and CSF IgA. (**Table 1**). SIRI was identified as a risk factor in the univariate analysis (**Table 2**). After excluding collinearity factors and after adjusting for other factors, multivariate analysis showed that SIRI was an independent factor to distinguish MOGAD from NMOSD (**Table 2**). It exhibited a high sensitivity and specificity at the cut-off value, and its elevated positive predictive value (PPV), positive likelihood ratio (PLR+), and diagnostic odds ratio (dOR) also indicated its diagnostic value (cut-off value = 1.565, sensitivity =0.68, specificity =0.70, PPV = 0.49, PLR+ =2.27, dOR = 5.04) (**Table 3**, **Figure 1A**). The ROC curve analysis indicated that CSF WCC had the highest specificity, PPV, PLR+, and dOR at the cut-off value (cut-off value = 17.5, specificity = 0.818, PPV = 0.58, PLR+ = 3.36, dOR = 7.00) (**Table 3**, **Figure 1A**). However, the univariate analysis showed that CSF WCC were not a risk factor (**Table 2**). We then analyzed the association between SIRI and other indicators in MOGAD patients and NMOSD patients, respectively. Spearman’s correlation analysis showed that SIRI was positively correlated with CSF WCC in MOGAD patients (p = 0.002, r = 0.667) and negatively correlated with CSF proteins and CSF IgG levels in NMOSD patients (p = 0.025, rs = -0.331; p = 0.011, rs = -0.379). When compared to HC, both MOGAD and AQP4-IgG-positive NMOSD presented significantly elevated neutrophils, NLR, SIRI, and SII (**Table 1**, **Figure 2**). ROC curve analysis indicated that all four of these indicators had excellent diagnostic performance for separating MOGAD from HC (**Figure 1B**).

**Table 2:** Indicators for distinguishing MOGAD from AQP4-IgG-positive NMOSD .

Variables	Univariate analysis			Multivariate analysis		
	OR	95% CI	P value	OR	95% CI	P value
NEU, 10^9^/L	1.206	1.048-1.389	**0.009**			
LYM, 10^9^/L	0.919	0.547-1.542	0.748			
MON, 10^9^/L	2.146	0.275-16.717	0.466			
PLT, 10^9^/L	1.006	0.998-1.014	0.152	1.000	0.992–1.009	0.937
NLR	1.059	0.987-1.135	0.111			
MLR	8.134	0.376-175.938	0.181			
SIRI, 10^9^/L	1.320	1.021-1.705	**0.034**	1.316	1.009–1.716	**0.043**
PLR	1.001	0.998-1.004	0.597			
SII, 10^9^/L	1.000	1.000-1.000	0.116			
CSF proteins, g/L	1.015	0.512-2.014	0.965			
CSF WCC, 10^6^/L	1.005	0.997-1.012	0.222			
CSF IgG, mg/L	1.002	0.995-1.009	0.574			
CSF IgA, mg/L	1.029	0.977-1.085	0.282			
CSF IgM, mg/L	1.023	0.924-1.133	0.658			

NEU, neutrophil; LYM, lymphocyte; MON, monocyte; PLT, platelet; NLR, neutrophil to lymphocyte ratio; MLR, monocyte to lymphocyte ratio; PLR, platelet to lymphocyte ratio; SIRI, systemic inflammation response index, monocyte × NLR; SII, systemic immune inflammation index, platelet × NLR; CSF, cerebrospinal fluid; WCC, white cell counts; IgG, immunoglobulin G; IgA, immunoglobulin A; IgM, immunoglobulin M; OR, odds ratio. bold, p<0.05.

**Table 3:** The ROC curve’s capacity to discriminate between MOGAD and AQP4-IgG-positive NMOSD.

Variables	AUC	95% CI	P value	Cut-off value	Sensitivity	Specificity	PPV	NPV	PLR+	NLR-	dOR
NEU, 10^9^/L	0.658	0.528-0.788	**0.022**	11.240	0.360	0.933	0.69	0.78	5.37	0.69	7.78
LYM, 10^9^/L	0.503	0.364-0.642	0.965								
MON, 10^9^/L	0.613	0.482-0.745	0.101								
PLT, 10^9^/L	0.599	0.472-0.725	0.154								
NLR	0.613	0.482-0.745	0.101								
MLR	0.624	0.494-0.755	0.072								
SIRI, 10^9^/L	0.692	0.567-0.818	**0.005**	1.565	0.680	0.700	0.49	0.85	2.27	0.45	5.04
PLR	0.554	0.424-0.685	0.432								
SII, 10^9^/L	0.623	0.497-0.750	0.074								
CSF proteins, g/L	0.644	0.511-0.776	0.057								
CSF WCC, 10^6^/L	0.667	0.499-0.834	**0.041**	17.500	0.611	0.818	0.58	0.86	3.36	0.48	7.00
CSF IgG, mg/L	0.609	0.471-0.747	0.157								
CSF IgA, mg/L	0.679	0.546-0.811	**0.021**	3.945	0.762	0.659	0.38	0.91	2.23	0.36	6.19
CSF IgM, mg/L	0.639	0.494-0.784	0.072								

NEU, neutrophil; LYM, lymphocyte; MON, monocyte; PLT, platelet; NLR, neutrophil to lymphocyte ratio; MLR, monocyte to lymphocyte ratio; PLR, platelet to lymphocyte ratio; SIRI, systemic inflammation response index, monocyte × NLR; SII, systemic immune inflammation index, platelet × NLR; CSF, cerebrospinal fluid; WCC, white cell counts; IgG, immunoglobulin G; IgA, immunoglobulin A; IgM, immunoglobulin M; AUC, area under the curve; PPV, positive predictive value; NPV, negative predictive value; PLR+, positive likelihood ratio; NLR-, negative likelihood ratio; dOR, diagnostic odds ratio. bold, p<0.05.

### 3.3 Factors predicting recurrence in patients with MOGAD

In the exploration of recurrent factors in MOGAD patients, we compared admission indicators between the 8 recurrent patients and the remaining 17 (**Table 4**). ROC curve analysis of the admission test results indicated MLR (AUC=0.805, 95% CI=0.616–0.994, cut-off value=0.200, sensitivity=0.750, specificity=0.882) was the only predictor for recurrence (**Figure 1C**). The other indicators were not statistically significant. In binary logistic regression analysis, MLR levels below 0.200 (odds ratio =22.5, 95% CI: 2.552–198.376, p = 0.005) were independently associated with recurrence. The PPV, negative predictive value (NPV), PLR+, negative likelihood ratio (NLR-), and dOR were 0.75, 0.88, 6.36, 0.28, and 22.71, respectively.

**Table 4:** Comparison of indicators of MOGAD patients with and without recurrence.

Variables	Recurrence(n=8)	Without recurrence(n=17)	P
Age, median, (IQR)	25(16.5,33.8)	27(14.5,38.5)	0.711
Female (n%)	3(37.5)	7(41.2)	1.000
EDSS, median, (IQR)	3.5(2.3,4.5)	3.0(1.5,4.3)	0.315
NEU, median, (IQR), 10^9^/L	7.70(5.43,12.46)	6.53(5.01,11.48)	0.628
LYM, median, (IQR), 10^9^/L	2.49(1.09,3.20)	1.75(1.00,2.30)	0.124
MON, median, (IQR), 10^9^/L	0.42(0.24,0.59)	0.41(0.35,0.65)	0.628
PLT, median, (IQR), 10^9^/L	268(226,310)	283(244,304)	0.842
NLR, median, (IQR)	2.71(1.92,12.91)	4.15(2.76,8.58)	0.406
MLR, median, (IQR)	0.18(0.14,0.26)	0.29(0.24,0.44)	**0.013**
SIRI, median, (IQR), 10^9^/L	1.35(0.95,3.75)	2.27(1.33,4.62)	0.215
PLR, median, (IQR)	107.74(88.02,246.13)	166.67(124.67,273.79)	0.124
SII, median, (IQR), 10^9^/L	731.90(478.84,3054.98)	1475.97(698.95,2493.39)	0.374
Imaging profile			
Cerebrum (without cortex) (n%)	4(50)	10(59)	>0.999
Cerebral cortex(n%)	2(25)	5(29)	>0.999
Optic nerve(n%)	2(25)	2(12)	0.570
Diencephalon(n%)	2(25)	0	0.093
Pons(n%)	2(25)	4(24)	>0.999
Medulla(n%)	0	1(6)	>0.999
Cerebellum(n%)	0	2(12)	>0.999
Cervical spinal cord(n%)	0	3(18)	0.527
Thoracic spinal cord(n%)	0	1(6)	>0.999
CSF profile			
Proteins, median, (IQR), g/L	0.44(0.26,0.61)	0.39(0.28,0.42)	0.731
WCC, median, (IQR), 10^6^/L	6(0,28)	38(2,110)	0.246
IgG, median, (IQR), mg/L	40.50(28.90,47.70)	35.40(26.45,59.70)	0.913
IgA, median, (IQR), mg/L	5.20(4.18,5.85)	5.05(3.07,11.80)	0.743
IgM, median, (IQR), mg/L	1.02(0.43,1.18)	0.55(0.26,2.96)	0.799
Treatment			
Steroid hormone(n%)	7(87.5)	10(58.8)	0.205
Steroid hormone + IVIG(n%)	1(12.5)	5(29.4)	0.624
Steroid hormone + IVIG + MMF(n%)	0	2(11.8)	>0.999

NEU: neutrophil; LYM: lymphocyte; MON: monocyte; PLT: platelet; NLR: neutrophil to lymphocyte ratio; MLR: monocyte to lymphocyte ratio; PLR: platelet to lymphocyte ratio; SIRI: systemic inflammation response index, monocyte × NLR; SII: systemic immune inflammation index, platelet × NLR; CSF: cerebrospinal fluid; WCC: white cell counts; IgG: immunoglobulin G; IgA: immunoglobulin A; IgM: immunoglobulin M; IVIG: intravenous immunoglobulin; MMF: mycophenolate mofetil. bold, p<0.05.

## 4 Discussion

Our findings show that MOGAD and AQP4-IgG-positive NMOSD are different illnesses, as evidenced by imaging, blood, and CSF data. MOGAD primarily affected the cerebrum and cerebral cortex, while the medulla, cervical spinal cord, and thoracic spinal cord were more likely to be observed in AQP4-IgG-positive NMOSD. Significant differences were also observed in SIRI, CSF WCC, and CSF IgA levels between MOGAD and AQP4-IgG-positive NMOSD.

SIRI is a useful indicator for predicting the prognosis of a number of inflammatory diseases. According to Topkan et al., a low SIRI score is associated with prolonged progression-free survival and overall survival in patients with glioblastoma multiforme of the brain (19). Yun et al. found that SIRI is an independent risk factor for poor outcomes in patients with aneurysmal subarachnoid hemorrhage (8). Zhang et al. discovered that SIRI is closely linked to stroke mortality and severity, as well as the risk of sepsis. The worse the stroke prognosis, the higher the SIRI value (5). In our study, we found that the MOGAD group had greater SIRI levels than the AQP4-IgG-positive NMOSD and HC groups. We identified a key SIRI threshold of 1.565 for distinguishing MOGAD from AQP4-IgG-positive NMOSD. Although clinical evidence on SIRI in demyelinating diseases is limited, further investigation is recommended for its potential clinical utility in MOGAD.

SIRI reflects complex interactions among neutrophils, monocytes, and lymphocytes. It’s worth mentioning that SIRI integrates monocytes, whereas SII incorporates platelets. Our study demonstrated that MOGAD patients had higher platelets and SII levels compared to AQP4-IgG-positive NMOSD patients, though without statistical significance. This implies that monocytes could contribute to the distinct SIRI disparity between MOGAD and AQP4-IgG-positive NMOSD. In experimental autoimmune encephalomyelitis (EAE), monocytes played a decisive role in disease onset and demyelination (9–11). Monocytes infiltrating the central nervous system (CNS), particularly Ly6ChiCCR2+ inflammatory monocytes, promoted EAE progression by crossing the blood-brain barrier in a CCR2-dependent manner (9, 20). Once inside the CNS, these cells released inflammatory mediators that accelerated disease development (20–22). Cost-effective and easily obtainable from blood samples, SIRI emerges as a novel inflammatory indicator for distinguishing MOGAD from AQP4-IgG-positive NMOSD.

MOGAD had considerably greater CSF WCC and CSF IgA levels than AQP4-IgG-positive NMOSD. Liu et al. found that C3 levels in the blood plasma were positively correlated with CSF WCC (23). In the MOGAD group, C3 consumption was lower, while CSF WCC were higher (23). This indirectly supports the earlier idea that the majority of MOG-IgG detected in the CSF is synthesized intrathecally by plasmablasts inside the CSF, whereas most of the AQP4-IgG in the CSF originated from extrathecal sources and passively entered the CSF through the blood-brain barrier (24).

MLR emerges as a predictor of MOGAD recurrence, implying that MOGAD patients with low MLR may require intensified treatment strategies. Previous research has shown that high MLR is correlated with poor prognosis in stroke patients and increased susceptibility to recurrence in AQP4-IgG-positive NMOSD patients (14, 25), in contrast to our findings of an association between lower MLR and MOGAD recurrence. These discrepancies potentially arise from functional disparities between neutrophils, monocytes, and lymphocytes across distinct diseases. Increasing evidence points to the detrimental role of neutrophils in CNS inflammation (26–29). Neutrophils have been detected in active lesions of both AQP4-IgG-induced NMO animal models and early-stage NMOSD patients (26, 30). Furthermore, damaged neutrophils in MOGAD and AQP4-IgG-positive NMOSD had distinct mechanisms of demise (28). Although neutrophils may have differences in the pathological aspects of these two diseases, no difference was found in the blood examinations of our patients.

Several limitations of our study deserve acknowledgment. Firstly, as a single-center investigation with only Han Chinese participants, it may introduce selection bias. The small sample size is another constraint of our study, which could limit our capacity to establish the potential significance of particular indicators. Additionally, the 12-month follow-up period might not fully capture disease dynamics in this demyelinating disorder. Future research should explore larger samples with a longer follow-up duration for more comprehensive insights. With more and more antibodies identified, some of the AQP4-IgG-negative NMOSD may be rectified as a new entity; hence, only AQP4-IgG-positive NMOSD patients were included in our study.

## 5 Conclusion

Our research found that increased SIRI may serve as indicators for distinguishing MOGAD from AQP4-IgG-positive NMOSD. Decreased MLR levels may be associated with the probability of MOGAD recurrence.

## Data availability statement

The raw data supporting the conclusions of this article will be made available by the authors, without undue reservation.

## Ethics statement

The studies involving humans were approved by The ethics committee of the Qilu Hospital of Shandong University (KLL2021-283). The studies were conducted in accordance with the local legislation and institutional requirements. Written informed consent for participation was not required from the participants or the participants’ legal guardians/next of kin in accordance with the national legislation and institutional requirements.

## Author contributions

LW: Conceptualization, Formal Analysis, Investigation, Methodology, Software, Visualization, Writing – original draft, Writing – review & editing. RX: Data curation, Formal Analysis, Software, Writing – review & editing. XL: Data curation, Formal Analysis, Software, Writing – review & editing. JS: Conceptualization, Formal Analysis, Project administration, Supervision, Validation, Writing – review & editing. SW: Conceptualization, Formal Analysis, Methodology, Project administration, Supervision, Validation, Writing – review & editing.
